# Color of the Eyes

**Published:** 1855-03

**Authors:** 


Color of the Eyes.—That the color of the eyes should affect their strength may seem strange; yet that such is the case need not at this time of day to be proved; and those whose eyes are brown or dark colored should be informed that they are weaker and more susceptible of injury, from various causes, than gray or blue eyes. Light blue eyes are *cæteris paribus*, generally the most powerful, and next to those are gray. The lighter the pupil, the greater and longer-continued is the degree of tension the eye can sustain.

